# Improving the Cellular Selectivity of a Membrane-Disrupting Antimicrobial Agent by Monomer Control and by Taming

**DOI:** 10.3390/molecules26020374

**Published:** 2021-01-13

**Authors:** Steven L. Regen

**Affiliations:** Department of Chemistry, Lehigh University, Bethlehem, PA 18015, USA; slr0@lehigh.edu

**Keywords:** antimicrobial, antibacterial, antifungal, antibiotic, membrane-disruption, drug design

## Abstract

Antimicrobial resistance represents a significant world-wide health threat that is looming. To meet this challenge, new classes of antimicrobial agents and the redesign of existing ones will be required. This review summarizes some of the studies that have been carried out in my own laboratories involving membrane-disrupting agents. A major discovery that we made, using a Triton X-100 as a prototypical membrane-disrupting molecule and cholesterol-rich liposomes as model systems, was that membrane disruption can occur by two distinct processes, depending on the state of aggregation of the attacking agent. Specifically, we found that monomers induced leakage, while attack by aggregates resulted in a catastrophic rupture of the membrane. This discovery led us to design of a series of derivatives of the clinically important antifungal agent, Amphotericin B, where we demonstrated the feasibility of separating antifungal from hemolytic activity by decreasing the molecule’s tendency to aggregate, i.e., by controlling its monomer concentration. Using an entirely different approach (i.e., a “taming” strategy), we found that by covalently attaching one or more facial amphiphiles (“floats”) to Amphotericin B, its aggregate forms were much less active in lysing red blood cells while maintaining high antifungal activity. The possibility of applying such “monomer control” and “taming” strategies to other membrane-disrupting antimicrobial agents is briefly discussed.

## 1. Antimicrobial Agents under Siege

According to a 2019 Antimicrobial Threats Report from the Centers of Disease Control and Prevention (CDC), more than 2.8 million antimicrobial-resistant infections occur each year in the United States, resulting in more than 35,000 deaths [1]. At present, the CDC considers the following bacteria as “urgent threats” to the public health: Carbapenem-resistant *Acinetobacter*, *Candida auris*, *Clostridioides difficle*, Carbapenem-resistant *Enterobacteriaceae*, and drug-resistant *Neisseria gonorrhoeae*. The CDC also considers the following 11 bacteria as “serious threats”: drug-resistant *Campylobacter*, drug-resistant *Candida*, ESBL-producing *Enterobacteriaceae*, Vancomycin-resistant *Enterococci* (VRE), multidrug-resistant *Pseudomonas aeruginosa*, drug-resistant nontyphoidal *Salmonella*, drug-resistant *Salmonella* serotype Typhi, drug-resistant *Shigella*, Methicillin-resistant *Staphylococcus aureus* (MRSA), drug-resistant *Streptococcus pneumoniae* and drug-resistant Tuberculosis. The two bacteria that the CDC views as “concerning threats” are Erythromycin-resistant Group A *Streptococcus* and Clindamycin-resistant Group B *Streptococcus*.

Although the world is now focusing on the development of vaccines and therapies to fight SARS-CoV-2, the coronavirus that is responsible for the current COVD-19 pandemic, the antimicrobial resistance problem (AMR) represents a major world-wide health threat that is looming [1,2,3,4,5,6,7,8,9,10,11]. While substantial resources have been made available to address the COVID-19 pandemic, relatively little investment is being made to combat the antimicrobial resistance problem. Because of a lack of financial incentive, this situation has been compounded by the abandonment of antimicrobial research by most of the large pharmaceutical companies. At present, the development of new antimicrobial agents is mainly being carried out in small start-up companies and academic laboratories with the financial support from venture capital, public, and philanthropic agencies. This emerging crisis of antimicrobial resistance and the withdrawal of the large pharmaceutical companies from research in this area has been discussed in a series of recent publications [5,6,7,8,9,10,11].

## 2. Why Consider Membrane-Disrupting Antimicrobial Agents?

Our own efforts in the antimicrobial area have been based on the belief that antimicrobial agents that operate at the membrane level are likely to be less susceptible to drug resistance than ones that must enter a microorganism to destroy it. Specifically, we reasoned that if an antimicrobial drug doesn’t have to enter the cytoplasm of a bacterial or fungal cell to kill it, then two of the more common mechanisms of drug resistance would be circumvented, i.e., enzymatic degradation and export mechanisms [12].

**Figure 1:**
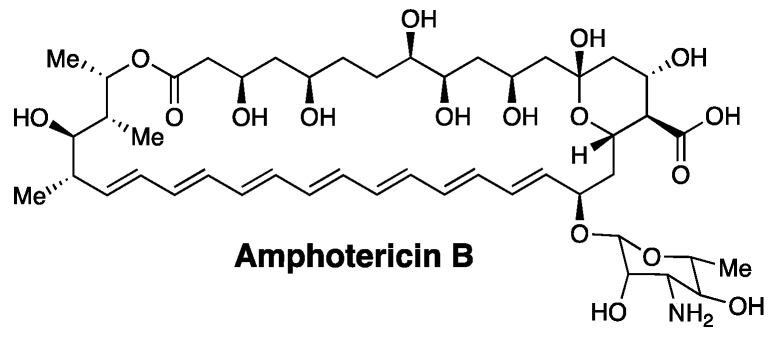
Structure of the Amphotericin B molecule.

A classic example of a membrane-disrupting antimicrobial agent that supports our thinking is the heptaene macrolide, Amphotericin B (Figure 1). Thus, despite its broad use in treating systemic fungal infections for more than 50 years, the development of resistance against this agent has proven to be extremely rare [13,14].

**Figure 2:**
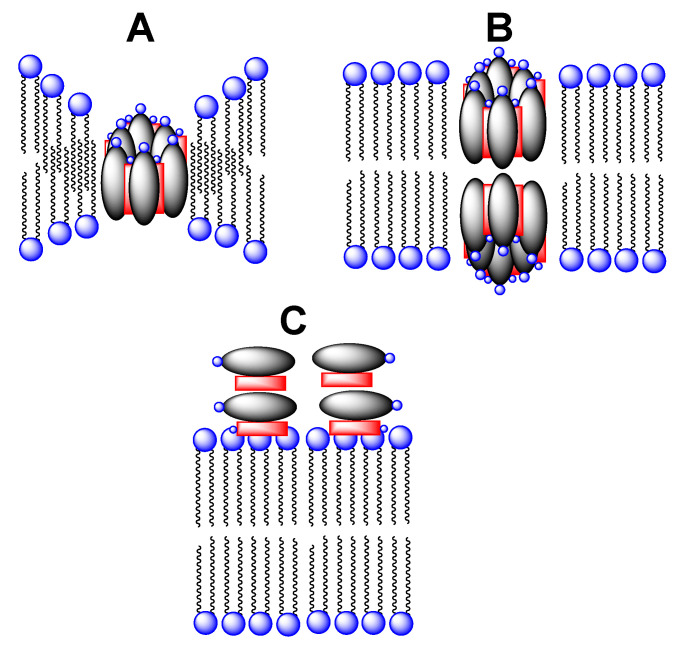
Stylized illustration showing (**A**) a single water-filled pore derived from Amphotericin B (gray oval) and ergosterol (red rectangle) with membrane thinning, (**B**) two aligned water-filled pores that extend across a lipid bilayer, and (**C**) a collection of Amphotericin B and ergosterol complexes lying on the surface of a fungal membrane.

Although there have been numerous studies of the membrane-disrupting action of Amphotericin B, the precise mechanism by which it destroys the integrity of the plasma membrane of fungal cells remains as a matter of debate. One of the oldest proposed mechanisms that remains popular is the “barrel stave” model (Figure 2) [15,16,17,18,19,20,21,22]. Here, Amphotericin B is thought to combine with ergosterol in fungal membranes to form water-filled pores through which monovalent ions (K^+^, Na^+^, H^+^, and Cl^−^) readily pass; the net result being cell death. Whether there is a thinning of the lipid membrane in the vicinity of individual pores, or whether two such pores must align across the membrane to form a contiguous channel remains as an alternate possibility.

More recently, an entirely different mechanism of action of Amphotericin B has been postulated. In this case, the heptaene macrolide is presumed to act like a “sponge” in removing ergosterol from fungal membranes and depositing it on the membrane’s surface (Figure 2C) [23]. The extent to which the barrel stave and sponge mechanisms may contribute to the overall antifungal activity of Amphotericin B, however, remains to be established.

## 3. The Issue of Toxicity

**Figure 3:**
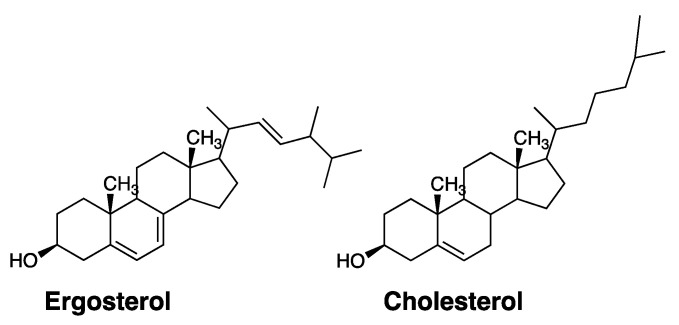
Molecular structures of ergosterol and cholesterol.

The value of Amphotericin B as an antifungal drug derives from its modest selectivity in destroying fungal cells over mammalian cells. This selectivity appears to be due to a higher affinity that Amphotericin B has towards ergosterol (the sterol found in fungi) over cholesterol (the sterol found in mammalian cells) (Figure 3) [17,22]. It should be noted, however, that despite its broad use in treating systemic fungal infections, Amphotericin B remains as one of the most toxic drugs that exists in modern medicine. Owing to its severe and potentially lethal side effects, clinicians often refer to Amphotericin B as “Ampho-terrible”.

The significant toxicity associated with Amphotericin B is, in fact, a common problem that exists for virtually all membrane-disrupting antimicrobial agents. To judge the toxicity of an agent to mammalian cells, it is common practice to first measure its hemolytic activity in vitro, i.e., its ability to release hemoglobin from red blood cells. In an enlightening study by Bolard and coworkers, it was reported that while aggregates of Amphotericin B exhibit both hemolytic and antifungal activity, its monomers exhibit only antifungal activity [24]. The basis for this striking difference, however, was not revealed in that study. It is noteworthy that this difference could account for the reduced toxicity that is associated with liposomal formulations of Amphotericin B (e.g., AmBisome), which are currently in clinical use. In particular, the liposomes may simply serve as a reservoir for releasing low concentrations of monomers of Amphotericin B [25].

## 4. Discovery of a Membrane Rupture and Leakage Dichotomy

**Figure 4:**
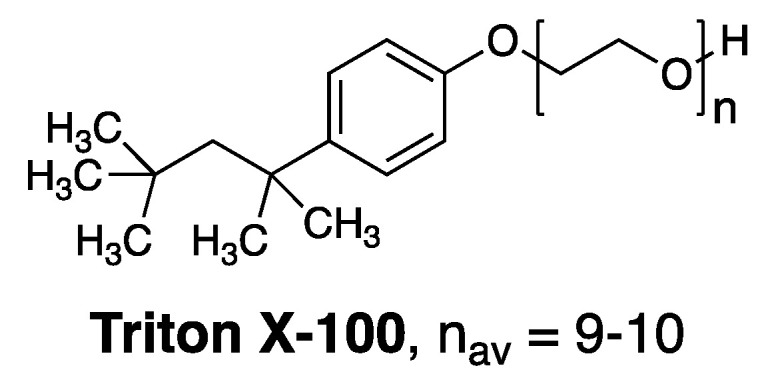
Structure of Triton X-100.

Prior to Bolard’s publication, we initiated a study that was aimed at gaining fundamental insight into how membrane-disrupting agents, in general, act on lipid bilayers [26]. In that study we focused on Triton X-100, a commonly used membrane-disrupting agent, as a prototype (Figure 4). A key discovery that we made was that Triton X-100 can disrupt cholesterol-rich bilayers by two distinct pathways depending upon its state of aggregation. Specifically, we found that below its critical aggregation concentration, attack by monomers resulted in the formation of leaky membranes. In sharp contrast, attack on similar cholesterol-rich membranes by aggregated forms of Triton X-100 (i.e., at concentrations in excess of its critical aggregation concentration), resulted in a catastrophic rupture of the membrane. These findings then led us to postulate that the hemolytic action of aggregated forms of Amphotericin B was the likely result of an analogous rupture events [26].

### 4.1. Separating Antifungal from Hemolytic Activity by Synthetic Design

**Figure 5:**
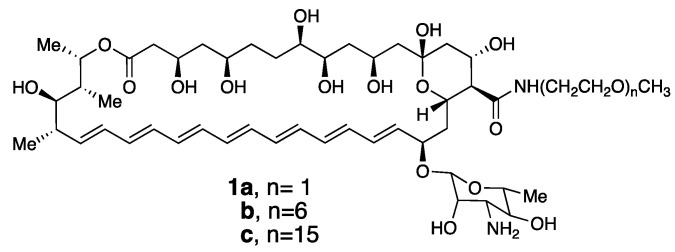
Poly(ethylene glycol) derivatives of Amphotericin B.

Based on our discovery of this membrane rupture and leakage dichotomy, we were motivated to synthesize a family of Amphotericin B derivatives in which their critical aggregation concentrations could be fined-tuned by adjusting the length of a pendant poly(ethylene glycol) unit, i.e., conjugates **1a**–**c** (Figure 5) [27].

**Figure 6:**
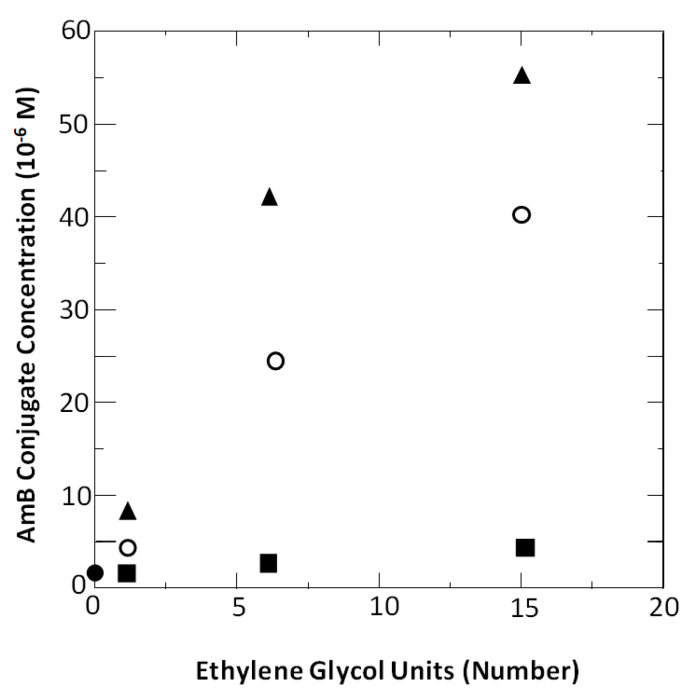
A plot of the number of ethylene glycol units in **1a**, **1b** and **1c** versus their (■) minimum concentration (MIC) needed to inhibit the growth of *Candida albicans*, (◯) critical aggregation concentration (cac), and (▲) concentration needed for 50% hemolysis (*K*_50_). One data point that is shown (●) represents the MIC, cac and *K*_50_ values for underivatized Amphotericin B. (Reprinted from [27]).

To our satisfaction, all three of these conjugates exhibited antifungal activity that was similar to that of the native Amphotericin B molecule. In addition, we found that the antifungal activity of each conjugate could be separated from its hemolytic activity. Specifically, significant hemolytic activity was observed only at concentrations that were in excess of their critical aggregation concentrations (Figure 6). These results were fully consistent with both Bolard’s findings and the rupture and leakage dichotomy that we discovered for Triton X-100.

### 4.2. A Taming Strategy

**Figure 7:**
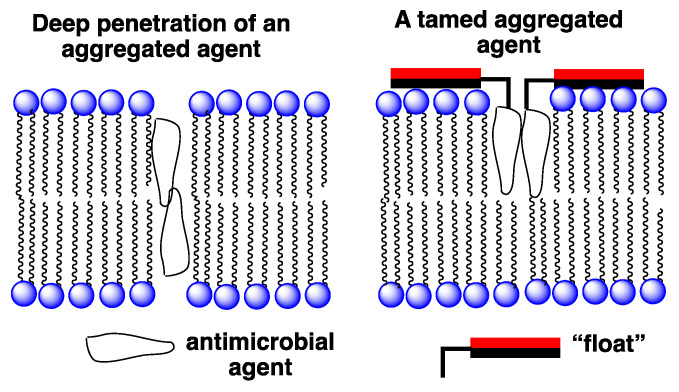
Illustration of: (**left**) an untamed aggregated agent extending across a lipid bilayer; (**right**) a tamed analog held close to the membrane’s surface. The red and black rectangles represent hydrophilic and hydrophobic faces of a facially amphiphilic moiety, respectively.

An entirely different approach that we have devised for improving the selectivity of membrane-disrupting antimicrobial agents in general, and for Amphotericin B in particular, is to reduce the hemolytic activity of aggregated forms via a “taming” strategy [28,29]. In Figure 7 an illustration is shown of our taming concept. Here, a membrane-disrupting antimicrobial agent is covalently attached to a molecule that serves as a “float”, i.e., a molecule that prevents deep penetration of the agent into the lipid bilayer. An example of such a float is the facially amphiphilic molecule, cholic acid, which is known to favor binding to the surface of lipid membranes [30]. Our working hypothesis was that by preventing such aggregates from extending across the membrane, rupture of the bilayer would prove difficult if not impossible.

### 4.3. The Taming of Amphotericin B

**Figure 8:**
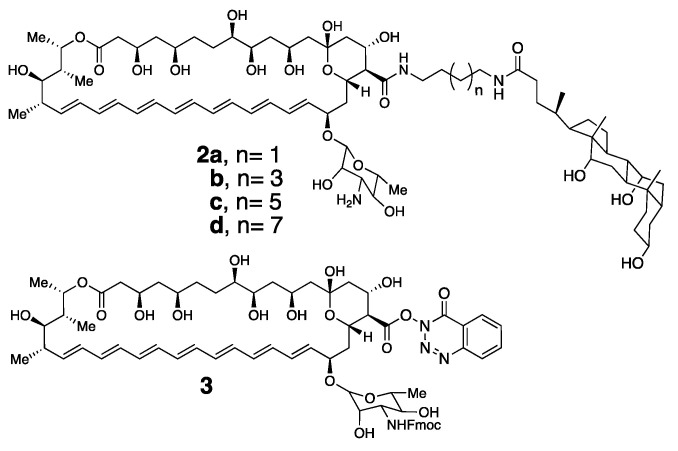
Molecular structure of tamed Amphotericin B molecules **2a**, **2b**, **2c** and **2d** and an activated and Fmoc-protected derivative of Amphotericin B, **3**.

For proof of concept, we synthesized a series of conjugates of Amphotericin B bearing one, and also two, choloyl moieties. In Figure 8, it is shown that the simplest representative examples in which Amphotericin B has been covalently attached to a single choloyl group using four different spacers, i.e., **2a**–**d** [29].

To prepare these conjugates, we first synthesized a derivative of Amphotericin B (i.e., **3**) in which its amino group was protected in the form of an Fmoc-carbamate, and its carboxylic acid moiety was activated by reaction with *N*,*N*,*N*′,*N*′-tetramethyl-*O*-(3,4-dihydro-4-oxo-123-benzotriazin-3-yl)uranium tetrafluoroborate [28]. This derivative has proven to be a very convenient and stable precursor for the synthesis of a broad series of conjugates of Amphotericin B. Thus, direct condensation of **3** with a family of α,ω-diamines that were monoacylated with cholic acid, followed by deprotection with piperidine afforded the desired conjugates, **2a**–**d** [29].

**Figure 9:**
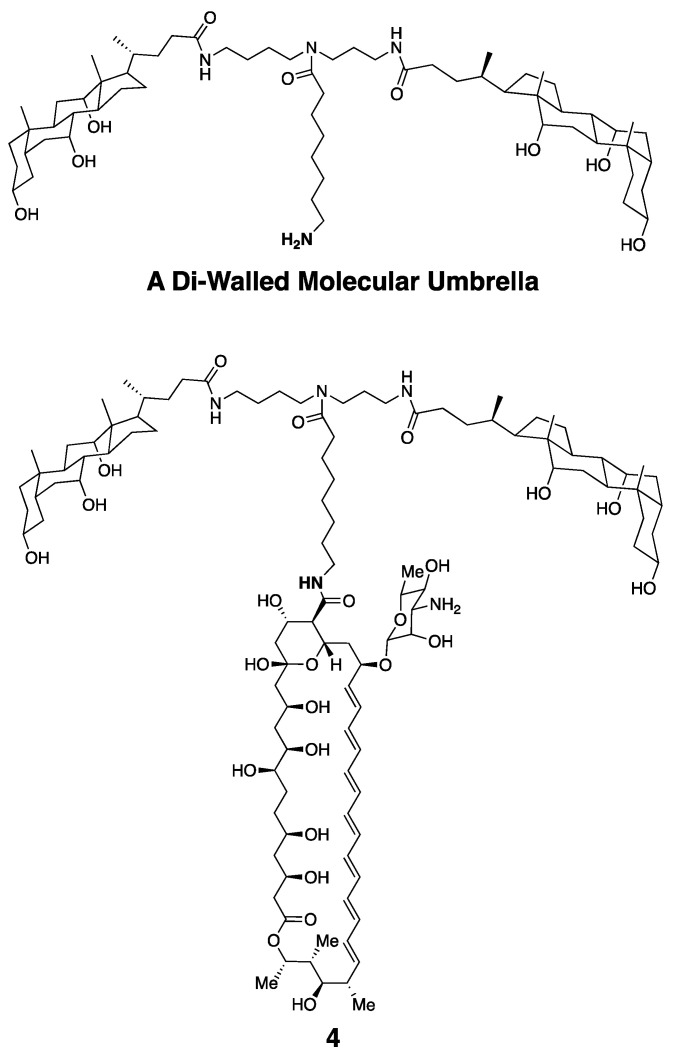
Molecular structure of a di-walled molecular umbrella and a derivative bearing the Amphotericin B molecule.

Taking advantage of **3**, and also a di-walled molecular umbrella bearing a pendant amine group, we could also readily synthesize an Amphotericin B conjugate bearing two choloyl moieties, **4** (Figure 9) [28].

It is noteworthy that in related studies we obtained evidence via a parallax analysis that an analog of **4**, bearing a pendant fluorophore (Cascade Blue), in place of Amphotericin B, favors binding to lipid membranes such that the choloyl moieties lie close to the membrane’s surface [31].

### 4.4. Aggregation Properties of Amphotericin B and Its Conjugates

To define the critical aggregation of Amphotericin B and its conjugates, we devised a simple approach that takes advantage of a well-defined absorption band for heptaene macrolide monomers, which lies at 409 nm [27]. Upon aggregation, the apparent molar absorptivity at 409 nm decreases. As discussed previously, if one defines ***T***, *m*, and *P* as the total, the monomeric and the aggregate concentrations of Amphotericin B, respectively, and if ***ε***, ***ε***_*m*_ and ***ε***_*p*_ are defined as the apparent molar absorptivity, the molar absorptivity of the monomer and the molar absorptivity of the aggregate components, respectively, then it can be shown that ***ε*** = ***ε***_*p*_ + (***ε***_*m*_ − ***ε***_*p*_)*m*/***T***. Thus, when concentrations Amphotericin B and its conjugates are in excess of their critical aggregation concentrations, *m* is a constant value and ***ε*** is expected to be inversely proportional to ***T***. Thus, by plotting the apparent molar absorptivity at 409 nm as a function of the reciprocal of the analytical concentration of Amphotericin B or a conjugate, one can estimate its critical aggregation concentration from the intercept of two straight lines. Based on this method, the critical aggregation concentration for **2a**–**d** and **4** were all estimated to be ca. 1 µM, which was the same as Amphotericin B, itself [28,29].

### 4.5. Separation of Antifungal from Hemolytic Activity via Taming

To evaluate the antifungal properties of **2a**–**d** and **4**, we determined their minimum inhibitory concentrations (MIC) with respect to *C. albicans*, *C glabrata*, *C. neoformans*, and *C. gatti* [28,29]. As shown in Table 1, the two conjugates having the shortest spacers, **2a**,**b**, exhibited broad-spectrum antifungal activities that compared favorably with Amphotericin B. Those analogs having longer spacers, **2c**, **d**, exhibited high antifungal activity against some but not all of the fungi that were tested. The conjugate bearing two choloyl moieties, **4**, also showed broad spectrum antifungal activities, which compared favorably with Amphotericin B.

To judge the toxicity of these membrane-disrupting antimicrobial agents towards mammalian cells, we evaluated their hemolytic activity using sheep red blood cells and determined the concentrations required for 50% hemolysis [29]. In the case of Amphotericin B, 50% hemolysis was observed using a concentration of 4 µM. In sharp contrast, the concentration of **2a** that was required for 50% hemolysis was 465 µM. For **2b**–**d**, negligible hemolysis was found at concentrations as high as 400 µM. With conjugate **4**, 50% hemolysis required a concentration of 375 µM. Thus, in all cases, these tamed derivatives of Amphotericin B were two or more orders of magnitude less hemolytic than the native drug.

To further evaluate the toxicity of these agents, we also examined their effect on HEK293 T cells. At a concentration of 25 µg/mL, Amphotericin B was highly toxic (<5% cell viability), **2a** was moderately toxic (ca. 40% cell viability), and **2b** was modestly toxic (ca. 70% cell viability). At this same 25 µg/mL, **2c** and **2d** showed close to negligible toxicity (>90% cell viability). In the case of conjugate **4**, no evidence of cytotoxicity was observed with a concentration as high as 100 µM (100% cell viability) [28].

### 4.6. Other Membrane-Disrupting Antimicrobial Agents Worthy of Consideration

In this review, I have discussed our fundamental studies with Triton X-100 that have led to a discovery of two distinct pathways of membrane disruption, i.e., mild leakage induced by monomers and catastrophic membrane rupture by aggregates. I have also described our design and synthesis of derivatives of the clinically important antifungal agent, Amphotericin B, which has allowed for the separation of the antimicrobial activity from the hemolytic activity by controlling their monomer concentration. A taming concept that I have discussed, in which facial amphiphiles are covalently attached to Amphotericin B to reduce its membrane rupturing activity is an entirely distinct approach for enhancing its cellular selectivity.

The generality of these two very different strategies (monomer control and taming) for improving the selectivity of other membrane-disrupting agents remains to be established. In the following, I briefly discuss, what I believe are, two broad classes of molecules whose therapeutic potential might be enhanced by either or both of these strategies.

**Antimicrobial Peptides**. Antimicrobial peptides (AMPs) represent a broad class of membrane-disrupting molecules that contain, typically 12 to 50 amino acids [32,33,34,35]. Since their discovery in the 1980′s, there has been considerable effort made to utilize naturally occurring AMPs, derivatives of AMPs and mimics of AMPs as drug candidates. A major problem with all of these agents, similar to Amphotericin B, is there low cellular selectivity and toxicity. In principle, if the selectivity of such molecules could be enhanced, this would aid in their development as therapeutic agents.

Closely related to antimicrobial peptides are, what have been termed, “cell-penetrating peptides” (CPPs) [36,37]. As previously noted, the difference in the functioning of antimicrobial peptides and cell-penetrating peptides may simply be a matter of the concentrations that are employed [38]. Thus, some peptides have already been shown to have both cell penetrating and lytic functions, and that these two distinct functions are strongly dependent on their concentration, i.e., at low concentrations they can penetrate cells, while at higher concentrations (where aggregation is likely) they lyse membranes [38,39]. Based on our studies with Triton X-100 and also Amphotericin B, it’s reasonable to believe that this behavior is directly related to the rupture and leakage dichotomy that we have discovered. To the best of my knowledge, careful studies that attempt to correlate membrane lysis and cell penetration with the aggregation properties of such peptides have not yet been reported. Given all of the research that has been made with antimicrobial and cell-penetrating peptides, I believe that such studies would be an important contribution that could afford new insight that helps to guide future work in this area.

**Quaternary Ammonium Compounds**. Because quaternary ammonium compounds are highly toxic to both mammalian cells and Gram-negative bacteria, their use as antimicrobial agents has been limited as disinfectants [40]. I believe that if attention were paid to the rupture and leakage properties of quaternary ammonium compounds, and if advantage could be taken of our monomer control and taming strategies, this could lead the way to new and useful therapeutic agents. It is noteworthy, in this regard, that a previous study of a series of quaternary ammonium compounds that were derived from L-phenylalanine were found to have a strong membrane selectivity dependence on their concentration [41]. Specifically, at high concentrations, where monomers and aggregates were present, these molecules showed significant hemolytic activity as well as antibacterial activity. In sharp contrast, at low concentrations where only monomers were present, these same molecules exhibited only antibacterial activity. Whether hybrid antimicrobial agents that are starting to emerge, (e.g., where quaternary ammonium groups are incorporated into potent antibiotics such as Polymyxin B and Vancomycin to aid in the destruction of bacterial membranes), could also take advantage of monomer control and taming is an intriguing question that, in my opinion, also warrants consideration [42,43].

## Figures and Tables

**Table 1:** Antifungal activities.

Microbe ^a^	AmB	2a	2b	2c	2d	4
*C. albicans*	0.5	1	2	2	>16	1
*C. glabrata*	0.5	2	2	>16	>16	2
*C. neoformans*	0.3	1	1	1	1	1
*C. gatti*	0.3	1	1	1	1	1

^a^ Minimum inhibitory concentrations (MIC) are the lowest concentrations (µg/mL) required for completely inhibiting fungal growth.

## Data Availability

All relevant data appear in the publications that are cited.
